# Interventions to treat and prevent postpartum depression: a protocol for systematic review of the literature and parallel network meta-analyses

**DOI:** 10.1186/s13643-022-02157-2

**Published:** 2022-12-28

**Authors:** David Thomas Monks, Basavaraj Ankalagi, Preet Mohinder Singh, Ebony Carter, Michelle Doering, Meg Guard, Shannon Lenze

**Affiliations:** 1 Department of Anesthesiology, Washington University School of Medicine, 660 S Euclid Avenue, St. Louis, USA; 2 Department of Anesthesiology, Toronto Western Hospital, University of Toronto, Toronto, Canada; 3 Department of Maternal and Fetal Medicine, Washington University School of Medicine, 660 S Euclid Avenue, St. Louis, USA; 4 Becker Library, Washington University School of Medicine, 660 S Euclid Avenue, St. Louis, USA; 5 Washington University School of Medicine, 660 S Euclid Avenue, St. Louis, USA; 6 Perinatal Behavioral Health Service, Department of Psychiatry, Washington University School of Medicine, St. Louis, USA

## Abstract

**Introduction:**

Postpartum depression has costly consequences for the mother, baby, and society. Numerous pharmacological and non-pharmacological interventions are available for the prevention and treatment of postpartum depression. To date, no attempt has been made to synthesize the evidence from comparisons of interventions both within and across these categories.

**Methods:**

We will perform a systematic review of the literature and perform network meta-analysis of interventions to (a) prevent and (b) treat postpartum depression. This review will include studies of primiparous or multiparous women during pregnancy or within 12 months of delivery of their baby that assess either interventions initiated during pregnancy or within 1 year of childbirth. Comparators will be other eligible interventions or control conditions. The outcome of interests will be related to the antidepressant efficacy of the interventions as well as their acceptability. The published literature will be searched in Ovid MEDLINE 1946-, Embase.com 1947-, Scopus 1823-, Cochrane Central Register of Controlled Trials, and ClinicalTrials.gov. The search will use a combination of standardized terms and keywords for postpartum depression, a sensitive search filter to limit for randomized controlled trials, and a librarian-created “humans” filter. The search results will be uploaded to the Covidence online systematic review platform (Veritas Health Information Ltd., Victoria, Australia) where two review team members will independently screen articles. We will extract data to include year of publication, language, country, participants (number, demographic data, eligibility criteria, psychiatric symptoms, and co-morbidities), characteristics of the intervention and control conditions, and reported outcomes. Risk of bias for each study will be assessed independently by two review authors using the RoB 2: A revised Cochrane risk of bias tool for randomized trials. Network meta-analysis will be performed using a Bayesian hierarchical model supplemented with a Markov chain Monte Carlo approach.

**Discussion:**

Postpartum depression is a devastating disease with long-lasting consequences. Given the numerous available interventions to both prevent and treat postpartum depression and the great number of studies comparing them, it is imperative that clinicians and patients are provided with an assessment of their comparative efficacy and acceptability.

**Systematic review registration:**

Prospero registration (CRD42022303247).

**Supplementary Information:**

The online version contains supplementary material available at 10.1186/s13643-022-02157-2.

## Introduction

Postpartum depression (PPD) complicates 6–19% of pregnancies, leading to costly consequences for the mother, baby, and society. The effects of PPD are long-lasting, with 25% of women continuing to have symptoms of depression 1 year after diagnosis, and 12.5% continuing to have symptoms for 2 years. Furthermore, of those women whose postpartum depression goes into remission, there is a 40% relapse rate [1, 2]. Unfortunately, suicidal thoughts are particularly common, affecting about 20% of women with PPD, and although still rare, suicide accounts for approximately 20% of postpartum deaths [3]. The societal burden is substantial: one study estimated an equivalent cost of $100,000 per case due to suicide, loss of work, morbidity, and infant malnutrition.

Numerous interventions are available for the prevention and treatment of postpartum depression. These include pharmacological, psychosocial, psychological, educational, and somatic therapies. To date, no attempt has been made to synthesize the evidence from comparisons of interventions both within and across these categories. Various systematic reviews of the literature have been published in the past decade, examining interventions to treat and prevent postpartum depression. These reviews have focused on specific interventions [4], groups of interventions [5] and specific patient groups [6] and have pooled data to perform pairwise meta-analysis or series of pairwise meta-analyses. For example, a 2021 Cochrane review [7] of antidepressant medications included 11 RCTs (1016 women) that compared antidepressants with placebo, treatment as usual, psychological interventions, psychosocial interventions, any other medicines, or another type of antidepressant; and complementary medicine (food supplements). They reported a series of meta-analyses between each of these and found that women treated with antidepressants might only experience a slightly better antidepressant benefit than women given a placebo. However, to our knowledge, there have been no network meta-analyses performed. Our planned analysis will attempt to compare all these interventions in a common network and utilize direct and indirect evidence to allow clinicians to compare their efficacy and safety. We aim to review the relevant literature and perform network meta-analysis of interventions to (a) prevent and (b) treat postpartum depression within 12 months of delivery. After reviewing our analysis, the reader should be able to assess the effectiveness of varying methods of preventing and treating PPD. Additionally, we will provide the reader with an understanding of the confidence in the evidence to support each intervention examined.

## Methods/design

A systematic review of the relevant literature will be performed, employing methods outlined in the Cochrane Handbook, and data extracted to permit network meta-analysis [8]. Separate networks will be constructed for (a) preventative and (b) treatment interventions. The degree of connection between the networks that predominantly compare pharmacological and non-pharmacological interventions will determine whether separate networks are necessary to calculate network estimates. This protocol has been reported in accordance with the Preferred Reporting Items for Systematic review and Meta-Analysis Protocols (PRISMA-P) guidelines [9]. The review will be reported in adherence with the PRISMA extension statement for incorporating network meta-analysis [10]. A completed PRISMA-P checklist for the current review protocol is provided in Supplementary Fig 1. This protocol was registered with PROSPERO (CRD42022303247).

### Eligibility criteria

Inclusion and exclusion criteria were defined according to the patient, intervention, comparator, outcomes, and study design format (PICOS).

#### Population

This review will include studies of primiparous or multiparous women during pregnancy or within 12 months of delivery of their baby. As the initial search will include studies for each of the review questions related to (a) prevention and (b) treatment of postpartum depression, participants may or may not be depressed at baseline. We will use the individual study definition of the intervention, as either preventative or therapeutic.

#### Interventions

Studies of both pharmacological and non-pharmacological interventions initiated during pregnancy or within 1 year of childbirth will be included. Pharmacological interventions will be categorized by class and further sub-categorized by individual drug and dosing regimen as justified by the data. These subcategories will be created to define separate interventions on the condition that there are sufficient comparisons with other interventions to permit a cohesive network. Similarly, non-pharmacological interventions will be broadly categorized based on the nature (psychological, psychosocial, somatic, physical therapy, sensory therapy, etc.) and sub-categorized by clustering of their characteristics. Combinations of interventions will be evaluated according to a similar data-driven strategy. In all instances, the data collected on such characteristics will either inform sub-categorization or be noted as a potential effect modifier and considered a candidate for subsequent meta-regression. A detailed description of the process of node definition and network construction will be reported with the results of this analysis. Table 1 provides an anticipated list of potential categories and subcategories of interventions.

**Table 1:** Examples of anticipated categories and potential subcategories of interventions

**Pharmacological**		
Fluoxetine	Citalopram	Levomilnacipran
Paroxetine	Escitalopram	Brexanolone
Nortriptyline	Mirtazapine	Ketamine
Venlafaxine	Desvenlafaxine	Bupropion
Nefazodone	Trazodone	Vilazodone
Fluvoxamine	Vortioxetine	Duloxetine
Sertraline		
**Psychosocial and psychological**		
Interpersonal therapy	Cognitive behavioral therapy	Problem solving treatment
Behavioral activation	Psychodynamic psychotherapy	Supportive psychotherapy
Acceptance and commitment therapy	Mindfulness-based stress reduction	
**Somatic therapies**		
Repetitive transcranial magnetic stimulation	Electroconvulsive therapy	
**Physical/sensory therapy**		
Acupuncture	Massage	Light therapy
Aerobic exercise	Yoga	Music therapy
**Educational**		
**Hormonal**		
**Social support**		

#### Comparators

In addition to the interventions described above, we anticipate a variety of control conditions which will be differentiated based on their broad categories (usual care, enhanced usual care, waiting-list, non-treatment, and placebo) and individual characteristics. We will, again, use this data and the integrity of potential networks to dictate the sub-division (or not) of these control conditions into separate nodes (interventions).

#### Outcomes

The outcome of interests will be related to the efficacy and acceptability of the interventions. Efficacy will be assessed, for preventative interventions, by difference in odds of developing PPD and, for therapeutic interventions, by odds of response. Response will be defined as the total number of patients who had a reduction of ≥ 50% of the total depression score, standardized from the validated depression scoring scale. Such rating scales include the Edinburgh Postpartum Depression Scale (EPDS), Hamilton Depression Rating Scale (HAMD), Beck Depression Inventory (BDI), Postpartum Depression Screening Scale, Patient Health Questionnaire (PHQ9), Self-Rating Depression Scale (SDS), Center for Epidemiologic Studies Depression Scale (CESD), Zung Self-Rating Depression Scale, Hospital anxiety and depression scale (HADS), and the Depression anxiety stress scale (DASS). Acceptability will be primarily assessed by modeling the odds of all-cause discontinuation of the interventions (the proportion of patients who withdrew for any reason). Additional outcomes will include related psychiatric symptoms and potential adverse effects of interventions such as anxiety, sedation, suicidal ideation, headache, nausea, dry mouth, insomnia, dizziness, diarrhea, constipation, sexual problems, fatigue, weight gain, tremors, and increased sweating. We will attempt to construct networks for any of these adverse effects that are consistently reported across enough studies and will report the rest in the table of study characteristics. Studies with crossover design will only be included if they give clear outcome data for each group (prior and after the cross over). Study groups evaluating mixed interventions will not be included in the evaluation unless the mixed intervention is considered as a separate node/intervention.

#### Study design

We will include all peer reviewed randomized and quasi-randomized clinical trials in our initial search. On appraisal of the available studies and the relative contribution of quasi-randomized comparisons, we will decide whether the benefit of including this data is outweighed by the consequent reduction in the confidence in the accumulated evidence.

### Search strategy and information sources

The published literature will be searched using strategies created by a medical librarian for clinical trials on postpartum depression. The search will be implemented in Ovid MEDLINE 1946-, Embase.com 1947-, Scopus 1823-, Cochrane Central Register of Controlled Trials, and Clinicaltrials.gov. The search will use a combination of standardized terms and keywords for postpartum depression, a sensitive search filter to limit for randomized controlled trials, and a librarian-created “humans” filter. Conference abstracts will be excluded from the Embase and Scopus searches. Results will be imported into Endnote and duplicates will be identified and removed. The search of ClinicalTrials.gov aims to identify emerging studies nearing completion. We will attempt to contact authors of any pertinent unpublished studies to ensure complete data extraction; however, abstracts will be excluded if data remains incomplete. Non-English publications will be included provided an English-language abstract is available. A draft search strategy of Ovid MEDLINE is provided in Table 2 and the strategies for the remaining databases can be found in Supplementary Table 1.

**Table 2:** Draft search strategy for Ovid MEDLINE

1	exp postpartum depression/ or Postpartum depression.mp. or maternal depression.mp. or post partum depression.mp. or post-partum depression.mp. or post-natal depression.mp. or post natal depression.mp. or postnatal depression.mp. or puerperal depression.mp. or puerperium depression.mp. OR (depression adj3 (postpartum OR postnatal)).mp.
2	((control adj3 group*) or ((patient or healthy or volunteer or volunteers) adj3 control*)).mp. or controlled clinical trial.pt. or (quasi* adj2 (randomiz* or randomis*)).mp. or randomized controlled trial.pt. or double-blind method/ or controlled clinical trials as topic/ or randomized controlled trials as topic/ or early termination of clinical trials as topic/ or (randomi?ed adj7 trial*).mp. or (double-blind adj1 method).mp. or (controlled adj3 trial*).mp. or ((single or doubl* or tripl* or treb*) and (blind* or mask*)).ti,ab,kw. or 4 arm.ti,ab,kw. or four arm.ti,ab,kw.
3	1 and 2
4	3 not ((exp Animals/ not (exp Animals/ and exp Humans/)) or rabbit.ti. or rabbits.ti. or rat.ti. or rats.ti. or cattle.ti. or bovine.ti. or mice.ti. or mouse.ti. or ovine.ti. or sheep.ti. or goat.ti. or dog.ti.)

### Study records

The search results will be uploaded to the Covidence online systematic review platform (Veritas Health Information Ltd., Victoria, Australia). Two review team members will screen articles independently using the predefined eligibility criteria. This will be done in two stages, the first by referring to title and abstracts only before confirming the eligibility decision by reference to the full text. In case of any disagreement, a third review team member will mediate consensus. Reasons for excluding full texts will be documented both in Covidence and an Excel spreadsheet. Study authors will be contacted if eligibility criteria remain unclear following article review. Final study inclusion will be presented in a PRISMA flow diagram.

### Data extraction

We will use six randomly selected studies of preventative and therapeutic interventions, respectively, to guide the development of a data extraction spreadsheet. In the event of an essential data item being identified beyond the initial six studies, we will reformat the data extraction tool and go back to extract this missing item from all trials. We will extract data to include year of publication, language, country, participants (number, demographic data, eligibility criteria, psychiatric symptoms, and co-morbidities), characteristics of the intervention and control conditions, and reported outcomes. In the prevention network, we will attempt to categorize the population in each trial as “asymptomatic, at risk”, “subclinical symptomatology” or “mixed asymptomatic/subclinical” in accordance with the recommendations of the Institute of Medicine’s report on prevention research [11]. We will also collect study-level data to inform the definition of interventions, as described above, and other sources of heterogeneity and effect modification.

### Assessment of quality of evidence

Risk of bias for each study will be assessed independently by two review authors using the RoB 2: A revised Cochrane risk of bias tool for randomized trials [12]. The overall risk of bias will be expressed as low risk, some concerns/uncertainty, or high risk. Publication bias will be assessed by visually inspecting a comparison-specific funnel plot (for the primary outcome). A funnel plot will be constructed for pairwise comparison for each of the treatment options included in our analysis. The Confidence in Network Meta-Analysis (CINeMA) approach will be used to evaluate the overall evidence quality. Trials will be individually assessed for the indirectness of evidence. Indirectness refers to the relevance of the included studies to the research question. It helps to establish how well the included studies address the research question for the present network meta-analysis. Included studies will be scored based on uniformity across three parameters: study participants, interventions, and outcome characteristics reported. The more divergence noted in these parameters, the more indirectness assumed. In addition, the GRADE tool will be employed to assess the certainty in the evidence for the pairwise comparison of each agent with a common comparator in the summary of findings table.

### Summary of findings table

For the primary outcome, a summary of findings table will be constructed. Summary of findings tables summarize the results of NMA, provide absolute estimates of each interventions’ effect when compared to a common comparator, report probability ranking and an assessment of the certainty in the evidence (using the GRADE tool) in order to facilitate comprehensive interpretation of the results of NMA. Once the network has been constructed, we will identify the intervention that is compared to the most different interventions (i.e., maximally connected) in the largest number of trials (maximal direct evidence) and use this as the common comparator for the purpose of the summary of findings table.

### Data synthesis

We will, initially, construct a network of the evidence to determine whether the data from comparisons of pharmacological and non-pharmacological interventions warrants separate analyses or whether one network can maximize the data available to inform network estimates. The resulting network meta-analysis/es will be conducted using Bayesian approach. The odds ratio and 95% CrI will be calculated for dichotomous outcomes. The mean difference (MD) and 95% credible intervals (CI) will be calculated for continuous outcomes. The geometry of each network will be reported in graphical form along with a league table of network estimates for each pairwise network estimates. We will calculate the cumulative probabilities for each intervention being at each possible rank and then use the surface under the cumulative ranking curve (SUCRA) to create a treatment hierarchy. SUCRA is a commonly used method to numerically summarize the cumulative rankings so that SUCRA is 1 when a treatment is certain to be the best and is 0 when a treatment is certain to be the worst [13]. Attempts will be made to locate and evaluate inconsistencies across the network using node-split modeling.

### Statistical analysis

Analysis will be performed using a Bayesian hierarchical model (binomial modeling with logit link function) supplemented with Markov chain Monte Carlo (MCMC) approach. We will initially run 5000 adaptations, 20,000 iterations with a thinning factor of 10. These parameters will be adjusted as necessary to achieve a Potential Scale Reduction Factor (PSRF) of less than 1.05. The convergence diagnostics for the model will be reported in Gelman Rubin diagrams. The indirect estimates will be imputed using common comparators. The outcomes will be reported as credible intervals (CrI). Based on the distribution of credible intervals, rank probabilities (preferred order of therapeutic success) will be calculated for all the included treatment nodes. The statistical analysis will be performed by R assisted by package “gemtc” (Version 0.8-7, Github.com), Netmeta (Version2.6-0, R-repository), Dmetar [14], and Bugsnet (bugsnetsoftware.github.io).

### Exploration of model fitness, transitivity, and inconsistency

Model fitness will be evaluated using the Deviance Information Criterion (DIC) values and overall deviance for each parameter analyzed. The model with the lowest DIC values (in comparison to the data points) will be used for reporting the results. We intend to use the following network global evaluation metrics to evaluate transitivity: deviance information criterion, net heat plot and direct evidence plot (although not evaluating transitivity, per se, the greater the contribution of direct evidence to each network estimate, the lower the likelihood of inconsistency). For each comparison, we shall also look at the node split model, thus helping us to quantify comparison-specific inconsistency to estimate deficiencies in transitivity. We have identified potential effect modifiers (Table 3) from the literature [15–18] and will assess the distribution of effect modifiers to judge if the transitivity assumption holds. For those that prove to be a study-level parameter, then we plan to explore the impact of this on our network estimates by employing network meta-regression. We plan to use both Bayesian and Frequentist tools available to localize and quantify the inconsistencies in the network. For this, we will construct a node-spit model and a net-heat plot. We will evaluate the proportion of direct comparisons in the final outcome using the direct evidence plot. Using this approach, we will estimate the minimum number of independent paths in the network contributing to the effect estimate at an aggregated level. “Minimum parallelism” and the “mean path length” will allow estimation of the degree of indirectness in the reported pooled outcome.

**Table 3:** A list of potential effect modifiers identified from the literature

a.Previous depression diagnosis [15–17]	
b.Lower education status [15]	
c.Unemployment [15]	
d.Low socioeconomic status [16]	
e.Low levels of social support [16]	
f.Advanced maternal age [17]	
g.Postpartum hemorrhage [17]	
h.Gestational diabetes [17]	
i.Gestational hypertension [17]	
j.Pre-eclampsia [17]	
k.Urgent cesarean [17]	
l.Preterm birth [17]	
m.Hyperemesis gravidarum [17]	

### Ethics and dissemination

Ethical approval was deemed unnecessary. We report here in a single manuscript, a broad and inclusive search strategy, designed to maximize the contribution of available evidence to answer two separate review questions: (a) what is the comparative effectiveness of available interventions to prevent postpartum depression? (b) what is the comparative effectiveness of available interventions to treat postpartum depression? We anticipate submitting these parallel network meta-analyses in separate manuscripts to recognized psychiatric journals.

## Discussion

Postpartum depression is a devastating disease with long-lasting consequences for patients, their families, and society. Given the numerous available interventions to both prevent and treat postpartum depression and the great number of studies comparing them, it is imperative that clinicians and patients are provided with an assessment of their comparative efficacy and acceptability along with a comprehensive appraisal of the quality of evidence that supports those assessments.

## Supplementary Information

**Additional file 1.** PRISMA-P (Preferred Reporting Items for Systematic review and Meta-Analysis Protocols) 2015 checklist: recommended items to address in a systematic review protocol*.

**Additional file 2: Supplemental Table 1.** Search strings.

## Data Availability

Not applicable.
