# Supplementary material for: Interventions to treat and prevent postpartum depression: a protocol for systematic review of the literature and parallel network meta-analyses
Source: Syst Rev. 2022 Dec 28;11:282. doi: 10.1186/s13643-022-02157-2 (PMC9798606; doi:10.1186/s13643-022-02157-2)
Supplement: Supplementary file 2 — Additional file 2: Supplemental Table 1. Search strings. [file 13643_2022_2157_MOESM2_ESM.docx]

Supplemental Table 1. Search strings

**Ovid Medline**

1 exp postpartum depression/ or Postpartum depression.mp. or maternal depression.mp. or post partum depression.mp. or post-partum depression.mp. or post-natal depression.mp. or post natal depression.mp. or postnatal depression.mp. or puerperal depression.mp. or puerperium depression.mp. OR (depression adj3 (postpartum OR postnatal)).mp.

2 ((control adj3 group*) or ((patient or healthy or volunteer or volunteers) adj3 control*)).mp. or controlled clinical trial.pt. or (quasi* adj2 (randomiz* or randomis*)).mp. or randomized controlled trial.pt. or double-blind method/ or controlled clinical trials as topic/ or randomized controlled trials as topic/or early termination of clinical trials as topic/or (randomi?ed adj7 trial*).mp. or (double-blind adj1 method).mp. or (controlled adj3 trial*).mp. or ((single or doubl* or tripl* or treb*) and (blind* or mask*)).ti,ab,kw. or 4 arm.ti,ab,kw. or four arm.ti,ab,kw.

3 1 and 2

4 3 not ((exp Animals/ not (exp Animals/ and exp Humans/)) or rabbit.ti. or rabbits.ti. or rat.ti. or rats.ti. or cattle.ti. or bovine.ti. or mice.ti. or mouse.ti. or ovine.ti. or sheep.ti. or goat.ti. or dog.ti.)

**Embase.com**

1. 'postnatal depression'/exp OR ‘Postpartum depression’:ti,ab,kw,de OR ‘maternal depression’:ti,ab,kw,de OR ‘post partum depression’:ti,ab,kw,de OR ‘post-partum depression’:ti,ab,kw,de OR ‘post-natal depression’:ti,ab,kw,de oR ‘post natal depression’:ti,ab,kw,de OR ‘postnatal depression’:ti,ab,kw,de OR ‘puerperal depression’:ti,ab,kw,de OR ‘puerperium depression’:ti,ab,kw,de OR (depression near/3 (postpartum OR postnatal)):ti,ab,kw

2. (control NEAR/3 group*):ti,ab,kw,de OR ((patient OR healthy OR volunteer OR volunteers) NEAR/3 control*):ti,ab,kw,de OR (quasi* near/2 (randomiz* OR randomis*)):ti,ab,kw,de OR 'controlled clinical trial':ti,ab,kw,de OR 'randomized controlled trial':ti,ab,kw,de OR 'double-blind method'/exp OR 'controlled clinical trials as topic'/exp OR 'randomized controlled trials as topic'/exp OR 'early termination of clinical trials as topic':ti,ab,kw,de OR (randomi?ed NEAR/7 trial*):ti,ab,kw,de OR ('double blind' NEAR/1 method):ti,ab,kw,de OR (controlled NEAR/3 trial*):ti,ab,kw,de OR ((single OR doubl* OR tripl* OR treb*):ti,ab,kw,de AND (blind* OR mask*):ti,ab,kw,de) OR '4 arm':ti,ab,kw,de OR 'four arm':ti,ab,kw,de

3. #1 AND #2

4. #3 NOT ([animals]/lim NOT [humans]/lim)

5. #3 NOT ([animals]/lim NOT [humans]/lim) AND ([article]/lim OR [article in press]/lim OR [data papers]/lim OR [editorial]/lim OR [erratum]/lim OR [letter]/lim OR [note]/lim OR [review]/lim OR [short survey]/lim)

**Scopus**

( ( TITLE-ABS-KEY ( "Postpartum depression" OR "maternal depression" OR "post partum depression" OR "post-partum depression" OR "post-natal depression" OR "post natal depression" OR "postnatal depression" OR "puerperal depression" OR "puerperium depression" ) ) ) AND ( ( TITLE-ABS-KEY ( control W/1 group ) ) OR ( TITLE-ABS-KEY ( randomi?ed W/4 trial* ) ) OR ( TITLE-ABS-KEY ( double-blind W/1 method ) ) OR ( TITLE-ABS-KEY ( controlled W/2 trial* ) ) OR ( TITLE-ABS-KEY ( ( single OR doubl* OR tripl* OR treb* ) AND ( blind* OR mask* ) ) ) ) AND NOT ( ( TITLE-ABS-KEY ( mouse ) ) OR ( TITLE-ABS-KEY ( rat ) ) OR ( TITLE-ABS-KEY ( monkey* ) ) OR ( TITLE-ABS-KEY ( pup ) ) OR ( TITLE-ABS-KEY ( rodent* ) ) OR ( TITLE-ABS-KEY ( dog* ) ) OR ( TITLE-ABS-KEY ( rats ) ) OR ( TITLE-ABS-KEY ( mice ) ) OR ( KEY ( animals ) ) ) AND NOT ( ( TITLE-ABS-KEY ( mouse ) ) OR ( TITLE-ABS-KEY ( animal-model* ) ) OR ( TITLE-ABS-KEY ( mice ) ) OR ( TITLE-ABS-KEY ( rats ) ) OR ( TITLE-ABS-KEY ( rat ) ) OR ( KEY ( animals ) ) AND ( KEY ( humans ) ) ) AND ( LIMIT-TO ( DOCTYPE , "ar" ) OR LIMIT-TO ( DOCTYPE , "re" ) OR LIMIT-TO ( DOCTYPE , "no" ) OR LIMIT-TO ( DOCTYPE , "ed" ) OR LIMIT-TO ( DOCTYPE , "le" ) OR LIMIT-TO ( DOCTYPE , "sh" ) OR LIMIT-TO ( DOCTYPE , "ch" ) OR LIMIT-TO ( DOCTYPE , "er" ) )

**Cochrane Central**

1. ‘Postpartum depression’:ti,ab,kw OR ‘maternal depression’:ti,ab,kw OR ‘post partum depression’:ti,ab,kw OR ‘post natal depression’:ti,ab,kw OR ‘postnatal depression’:ti,ab,kw OR ‘puerperal depression’:ti,ab,kw OR ‘puerperium depression’:ti,ab,kw

2. (quasi* near/2 (randomiz* OR randomis*)):ti,ab,kw OR (randomized NEAR/7 trial*):ti,ab,kw OR (randomised NEAR/7 trial*):ti,ab,kw OR ('double blind' NEAR/1 method):ti,ab,kw OR ((single OR doubl* OR tripl* OR treb*) near/7 (blind* OR mask*):ti,ab,kw) OR '4 arm':ti,ab,kw OR 'four arm':ti,ab,kw

3. #1 AND #2

**Clinicaltrials.gov**

(blinded OR randomized OR randomised OR control) AND AREA[ConditionSearch] postpartum depression
